# Hypoxia-cleavable and specific targeted nanomedicine delivers epigenetic drugs for enhanced treatment of breast cancer and bone metastasis

**DOI:** 10.1186/s12951-023-01939-7

**Published:** 2023-07-12

**Authors:** Zhaofeng Li, Peixin Liu, Wei Chen, Xueying Liu, Fan Tong, Junhui Sun, Yang Zhou, Ting Lei, Wenqin Yang, Dong Ma, Huile Gao, Yi Qin

**Affiliations:** 1 Department of Orthopedic, Zhuhai People’s Hospital (Zhuhai hospital affiliated with Jinan University, Zhuhai, 519000 Guangdong China; 2 Key Laboratory of Drug-Targeting and Drug Delivery System of the Education Ministry and Sichuan Province, Sichuan Engineering Laboratory for Plant-Sourced Drug and Sichuan Research Center for Drug Precision Industrial Technology, West China School of Pharmacy, Sichuan University, Chengdu, 610041 China; 3 Department of Biomedical Engineering, Jinan University, Guangzhou, 510632 China; 4 Department of Orthopaedic Surgery, Third Hospital of Hebei Medical University, Shijiazhuang, China

**Keywords:** Bone metastasis, Epigenetic drug, Nanomedicine, Drug delivery, Hypoxia responsive

## Abstract

Breast cancer bone metastasis has become a common cancer type that still lacks an effective treatment method. Although epigenetic drugs have demonstrated promise in cancer therapy, their nontargeted accumulation and drug resistance remain nonnegligible limiting factors. Herein, we first found that icaritin had a strong synergistic effect with an epigenetic drug (JQ1) in the suppression of breast cancer, which could help to relieve drug resistance to JQ1. To improve tumor-targeted efficacy, we developed a hypoxia-cleavable, RGD peptide-modified poly(D,L-lactide-co-glycolide) (PLGA) nanoparticle (termed ARNP) for the targeted delivery of JQ1 and icaritin. The decoration of long cleavable PEG chains can shield RGD peptides during blood circulation and reduce cellular uptake at nonspecific sites. ARNP actively targets breast cancer cells via an RGD-αvβ3 integrin interaction after PEG chain cleavage by responding to hypoxic tumor microenvironment. In vitro and in vivo assays revealed that ARNP exhibited good biodistribution and effectively suppressed primary tumor and bone metastasis. Meanwhile, ARNP could alleviate bone erosion to a certain extent. Furthermore, ARNP significantly inhibited pulmonary metastasis secondary to bone metastasis. The present study suggests that ARNP has great promise in the treatment of breast cancer and bone metastasis due to its simple and practical potential.

**Supplementary Information:**

The online version contains supplementary material available at 10.1186/s12951-023-01939-7.

## Introduction

Breast cancer has become the most common cancer in the world and greatly affects women’s health [1]. Bone metastasis occurs in up to 60–80% of patients with advanced breast cancer [2], further exacerbating their poor clinical outcomes [3]. Breast cancer cells often metastasize to the spine and long bones of the extremities, which easily leads to bone-related events such as nerve compression, hypercalcemia, and pathological fractures [4]. In addition, the interaction of breast cancer cells and the bone microenvironment increases the ability of cancer cells to re-metastasize to other organs, such as the lung, significantly shortening patient survival [5]. Bone metastasis is relatively easier to manage than visceral metastasis [6]. The current treatment of bone metastasis includes chemotherapy, endocrine therapy, surgery and external beam radiotherapy [7]. Chemotherapy is the cornerstone of bone metastasis treatment. However, due to the complex content of breast cancer bone metastasis, chemotherapy also faces enormous challenges, including drug resistance, insufficient local accumulation and systemic side effects. Furthermore, chemotherapy drugs often lead to abnormal bone metabolism [8], exacerbating the progression of bone-related events. Therefore, it is important to develop an anti-bone metastatic strategy with efficient targeted delivery and improved bone mass.

Epigenetic modification plays an instrumental role in the regulation of cancer oncogene activity and expression and is closely involved in cancer development and metastasis. Bromodomain and extra terminal domain (BET) family proteins (BRD2, BRD3, BRD4, and BRDT) are epigenetic readers that recognize acetylated lysines on histone tails and transcription factors, which are involved in chromatin remodeling and transcriptional activation [9]. BET proteins participate in the development of multiple cancers by driving the transcription of key oncogenes [10]. JQ1, a BET bromodomain inhibitor, can inhibit the expression of these oncogenes and cell proliferation by utilizing competitive binding to displace BET proteins from acetylated histones [11, 12]. Recently, JQ1 has shown promising results in tumor therapy. For example, treatment with JQ1 can significantly inhibit the proliferation of triple-negative breast cancer [13]. JQ1 reduces the viability of osteosarcoma cells and inhibits the differentiation of osteoclasts, thereby breaking the vicious cycle between bone tumor and bone resorption [14]. However, cancer cells can develop resistance to JQ1 through multiple mechanisms [15]. It has been shown that effective and reasonable drug combination could increase the antitumor efficacy and decrease the drug-resistance in tumors [16, 17]. Icaritin is a prenylflavonoid derivative from the Epimedium genus that has a variety of pharmacological and biological functions. Previous studies have demonstrated that icaritin inhibits the proliferation and differentiation of osteoclasts and promotes the function of osteoblasts, thereby treating osteoporosis [18]. Additionally, icaritin exerts anti-breast cancer effects by inhibiting the cell cycle and promoting apoptosis [19]. However, whether JQ1 and icaritin have a synergistic effect in breast cancer bone metastasis treatment is currently unclear.

Epigenetic drugs regulate gene expression via action on epigenetic enzymes and offer great promise as cancer treatments. They can directly inhibit cancer cell growth [20], enhance antitumour immunity [21] and regulate bone-related cell properties [22]. For example, epigenetic drug EPZ inhibits the expression of EZH2, thereby inhibiting the secondary metastasis from bone lesions in breast cancer [6]. However, these novel drugs also have dose-limiting toxicities and nonspecific distribution problems, resulting in poor tumor treatment efficacy [23]. In recent years, the development of nanomedicine has provided new directions and strategies for improving drug delivery [24, 25]. For instance, the co-delivery of doxorubicin and H4R4 peptides by using poly (ethylene glycol)-mediated metal-organic frameworks can significantly suppress breast cancer growth [26]. Bone-targeted micelles loaded with bortezomib for the treatment of breast cancer bone metastasis showed the advantages of low toxicity and enhanced therapeutic efficacy [27]. There are several delivery strategies in the treatment of bone metastasis, including nontargeted drug delivery, bone-targeted drug delivery and cancer cell-targeted drug delivery [3, 28, 29]. Even though these strategies help to enhance drug delivery efficacy, they also face the limiting factor that needs to be improved [30]. For instance, passive targeted drug delivery relies on the EPR effect but lacks active tumor uptake. Bone-targeted delivery is based on the specific binding of bisphosphonates (BPs) to hydroxyapatite (HAP) to deliver the drug, but the high binding ability of BPs to HAP also allows normal bone tissue to take up the drug, thereby affecting normal bone metabolism. Using receptors highly expressed by tumor cells and modifying corresponding ligands on nanoparticles can achieve active cancer cell targeting, but these receptors are also expressed in normal cells, resulting in nonspecific uptake of nanoparticles. Therefore, designing a new delivery system to overcome the deficiencies of previous strategies has practical application significance. Bone metastasis has specific characteristics due to the special anatomical microstructure and function of bone. αvβ3 integrin is highly expressed in breast cancer cells, and a previous study confirmed that bone-metastatic breast cancer cells expressed the integrin β3 subunit more strongly than primary breast cancer cells [31, 32]. Additionally, the oxygen partial pressure inside the bone tissue is lower than that of the peripheral tissue under normal physiological conditions, and the tumor tissue is also hypoxic [33, 34]; thus, it can be speculated that the degree of hypoxia at the bone tumor site is more severe. Considering that αvβ3 is also expressed in normal tissues and the hypoxic properties of bone metastasis, we tried to combine the EPR effect and active targeting ability to construct a novel but practical drug delivery system.

In this study, we first demonstrated that icaritin and one epigenetic drug, JQ1, had an effective synergistic role in inhibiting breast cancer cells in vitro. After that, we constructed two linear chimeric molecules, DSPE-PEG2000-RGD and DSPE-Azo-mPEG5000, by utilizing RGD with αvβ3 integrin binding property and hypoxia-responsive azobenzene (Azo) as the main functional groups. In the presence of poly(D,L-lactide-co-glycolide) (PLGA), these two amphiphilic molecules could encapsulate JQ1 and icaritin and self-assemble into micelles (termed ARNP). The decoration of a long cleavable PEG chain prolongs the circulation time of ARNP and thus enhances the EPR effect. Meanwhile, long cleavable PEG chains can shield RGD peptides during the circulation process, reducing nanoparticle uptake by normal cells. Following passive accumulation of ARNP at tumor sites, long PEG chains can be cleaved in response to the hypoxic tumor microenvironment, thereby exposing RGD peptides. The interaction of RGD-αvβ3 integrin specifically promotes the uptake of ARNP by breast cancer cells, thus achieving active targeting. Moreover, JQ1 and icaritin encapsulated in ARNP are released into the tumor, exerting a synergistic antitumor effect. In summary, we evaluated the distribution and antitumor effect at both the cellular and animal levels and provided insights into the potential ability of ARNP to treat breast cancer and bone metastasis.

**Scheme 1:**
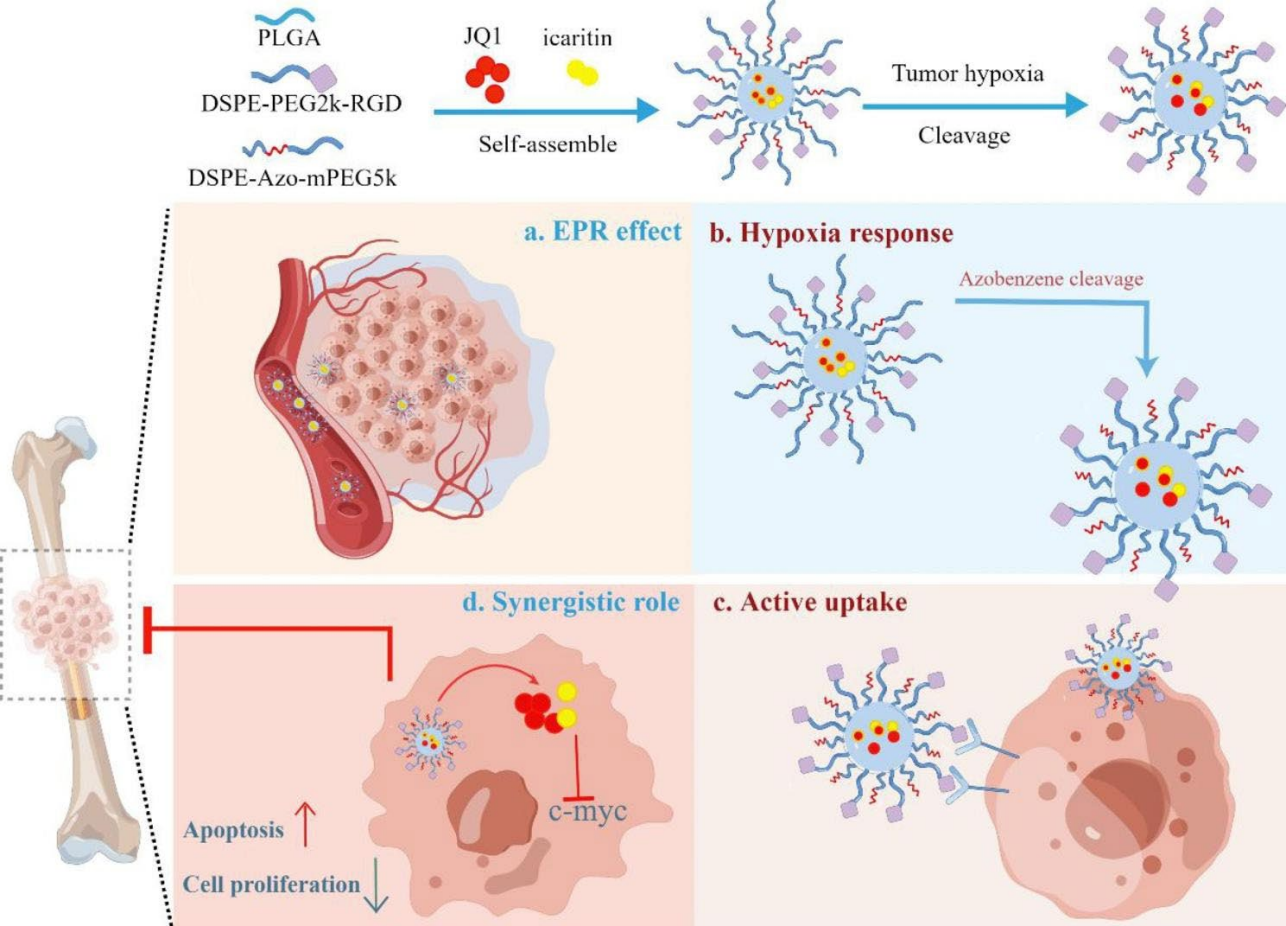
Schematic illustration of the composition of ARNP and its therapeutic effect on primary breast cancer and bone metastasis

## Results and discussion

### The combination of JQ1 and icaritin effectively inhibits breast cancer cells

**Fig. 1:**
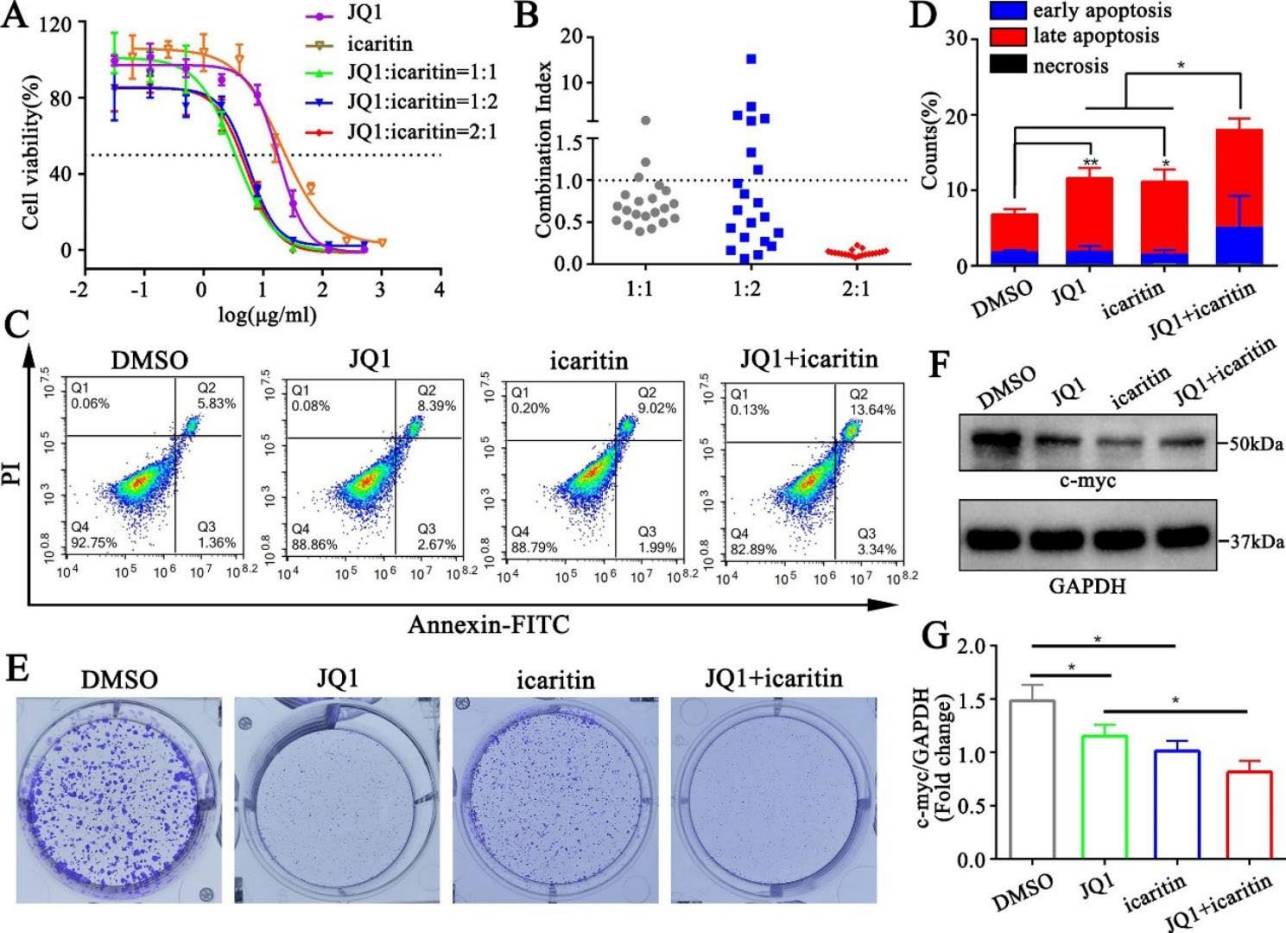
The combined effect of JQ1 and icaritin in inhibiting breast cancer cells. (**A**) MTT assay of 4T1 cells treated with JQ1 and icaritin (n = 6). (**B**) The combination index of JQ1 and icaritin at different concentration ratios (n = 6). (**C**) Apoptosis analysis of 4T1 cells treated with JQ1 and icaritin (n = 3). (**D**) Quantitative analysis of the apoptosis of 4T1 cells treated with different drugs (n = 3). (**E**) Colony formation assay of 4T1 cells treated with JQ1 and icaritin (n = 3). **F** and **G**) Protein expression of c-myc was determined by western blotting analysis (n = 3). All data are presented as the mean ± SD. *p < 0.05, **p < 0.01

To verify the synergistic effect of JQ1 and icaritin, we first conducted an MTT assay to determine the drug’s inhibitory effect on the breast cancer cell line 4T1. The MTT results showed that the IC_50_ values of JQ1 and icaritin were 18.50 µg/ml and 20.62 µg/ml, respectively, while the IC_50_ values of the two drug combinations were significantly decreased to 3.4 µg/ml, 5.7 µg/ml and 5.3 µg/ml at JQ1 to icaritin ratios of 1:1, 1:2 and 2:1, respectively (Fig. 1A). The 2:1 ratio showed the lowest combination index (CI), indicating the strongest synergistic effect (Fig. 1B). Therefore, a concentration ratio of 2:1 was selected for subsequent experiments. Using Annexin V-FITC and PI double staining, we found that the early apoptosis and late apoptosis percentages of 4T1 cells treated with JQ1 + icaritin were higher than those of 4T1 cells treated with JQ1 or icaritin (Fig. 1C, D), suggesting that the combination treatment enhanced apoptosis of breast cancer cells. Then, we performed colony formation assays to reveal the proliferation of 4T1 cells treated with JQ1 or icaritin. The results demonstrated that 4T1 cells exhibited a significant reduction and moderate reduction in colony formation after treatment with JQ1 and icaritin, respectively. However, 4T1 cells hardly formed colonies when treated with JQ1 + icaritin, indicating that JQ1 and icaritin have a strong synergistic effect in inhibiting the proliferation of breast cancer cells (Fig. 1E). Previous studies have shown that c-myc plays an important role in cell apoptosis and cell proliferation and thus participates in the development of breast cancer [35], while JQ1 also inhibits the expression of c-myc [36]. Therefore, we wondered whether JQ1 and icaritin play a synergistic role by affecting the expression of c-myc. Western blotting results showed that after treatment with JQ1 or icaritin, the expression of c-myc was significantly reduced compared with that in the DMSO group (Fig. 1F, G). Furthermore, after simultaneous treatment with JQ1 and icaritin, c-myc expression was decreased significantly compared with that in the JQ1 group, indicating the synergistic inhibition of the combined drug. Thus, these results revealed that JQ1 and icaritin have a strong synergistic effect in suppressing breast cancer cells.

### Synthesis and characterization of hypoxia-cleavable active targeted nanoparticles

**Fig. 2:**
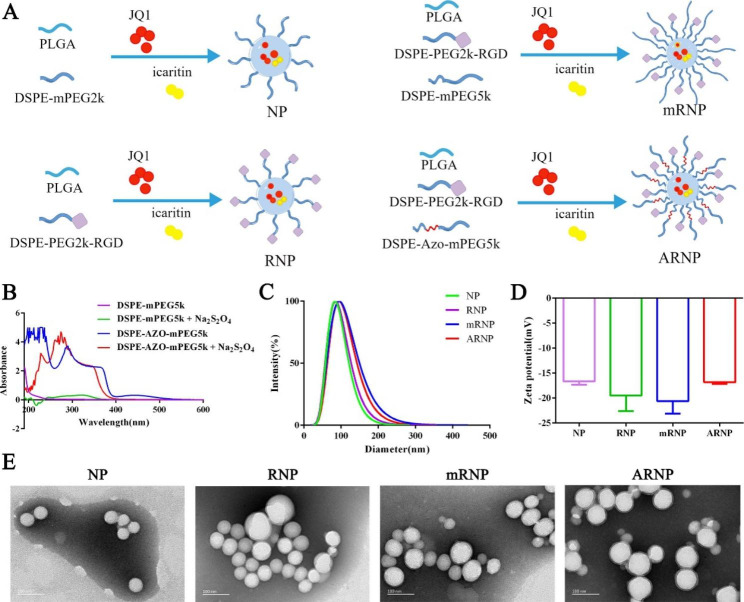
Synthesis and characterization of hypoxia-cleavable active targeted nanoparticles. (**A**) Schematic diagram of different nanoparticle structures. (**B**) UV‒Vis spectra of DSPE-Azo-mPEG5000 before and after hypoxic treatment. (**C**) The hydrodynamic diameters of NP, RNP, mRNP and ARNP. (**D**) The average zeta potentials of NP, RNP, mRNP and ARNP. (**E**) TEM images of NP, RNP, mRNP and ARNP. All data are presented as the mean ± SD (n = 3)

To improve the delivery efficiency of JQ1 and icaritin to tumor tissues, PLGA nanoparticles were selected as the drug delivery carrier. We first synthesized DSPE-PEG_2000_-RGD via the maleimide-thiol coupling reaction. The proton nuclear magnetic resonance (^1^ H-NMR) spectrum of CRGD showed critical peaks distributed from δ_H_ 8.00 to δ_H_ 8.71 (Supplementary Figure S2), which were assigned to the N-terminal amino group. The spectrum of DSPE-PEG2000-Mal showed critical peaks of polyethylene glycol at approximately δ_H_ 3.42 (Supplementary Figure S3), while all the critical peaks mentioned above existed in the spectrum of DSPE-PEG2000-RGD (Supplementary Figure S4), indicating successful synthesis. The matrix-assisted laser desorption/ionization time of flight mass spectrometry (MALDI-TOF-MS) showed peaks at m/z 2228.5 and m/z 2558.9, which belong to DSPE-PEG_2000_-Mal and DSPE-PEG_2000_-RGD, respectively (Supplementary Figure S5), further confirming the successful synthesis of DSPE-PEG_2000_-RGD. Then, we analyzed the synthesis of hypoxia-responsive DSPE-Azo-mPEG_5000_ by ^1^ H-NMR spectroscopy. The spectrum of azobenzene showed critical peaks of double benzene at approximately δ_H_ 8.08 (Supplementary Figure S6), while the critical peaks mentioned above existed in the spectrum of DSPE-Azo-mPEG_5000_ (Supplementary Figure S7), indicating successful synthesis. Due to their amphiphilic nature, PLGA and DSPE-PEG chains could self-assemble in aqueous conditions to form JQ1-icaritin-loaded nanoparticles. As shown in Fig. 2A, NP represents nontargeting drug-loaded nanoparticle, and RNP represents active targeting drug-loaded nanoparticle. mRNP represents active targeting and noncleavable drug-loaded nanoparticle, while ARNP means active targeting and hypoxia-cleavable drug-loaded nanoparticle. To evaluate the hypoxia-responsive cleavage of DSPE-Azo-mPEG_5000_, we used Na_2_S_2_O_4_, a classic deoxidizer, to mimic hypoxic conditions in vitro and applied UV‒Vis spectra to detect the characteristic peak of azobenzene. DSPE-Azo-mPEG_5000_ showed the characteristic peak at 450 nm, while it disappeared after treatment with Na_2_S_2_O_4_ (Fig. 2B), indicating azobenzene cleavage under hypoxic conditions. The hydrodynamic diameters of NP, RNP, mRNP and ARNP were 98.56 ± 6.89 nm, 99.83 ± 5.29 nm, 105.62 ± 7.91 nm and 108.39 ± 5.82 nm, respectively (Fig. 2C). The average zeta potentials of NP, RNP, mRNP and ARNP were − 16.66 ± 0.38 mV, -19.50 ± 1.81 mV, -20.62 ± 1.45 mV and − 16.85 ± 0.17 mV, respectively (Fig. 2D). The transmission electron microscopy (TEM) images revealed that the morphology of the prepared nanoparticles was generally spherical and uniformly dispersed (Fig. 2E). The encapsulation efficiencies of JQ1 and icaritin in different nanoparticles were approximately 85%, while the drug loading efficiencies of JQ1 and icatitin were all close to 15% and 7.5%, respectively (Supplementary Table S1). The stability measurements of the developed nanoparticles in 10% FBS were detected by dynamic light scattering and showed no obvious changes (Supplementary Figure S8), indicating their good stability in a physiological environment.

### ARNP shows promising cellular uptake and anticancer efficacy under hypoxic conditions

**Fig. 3:**
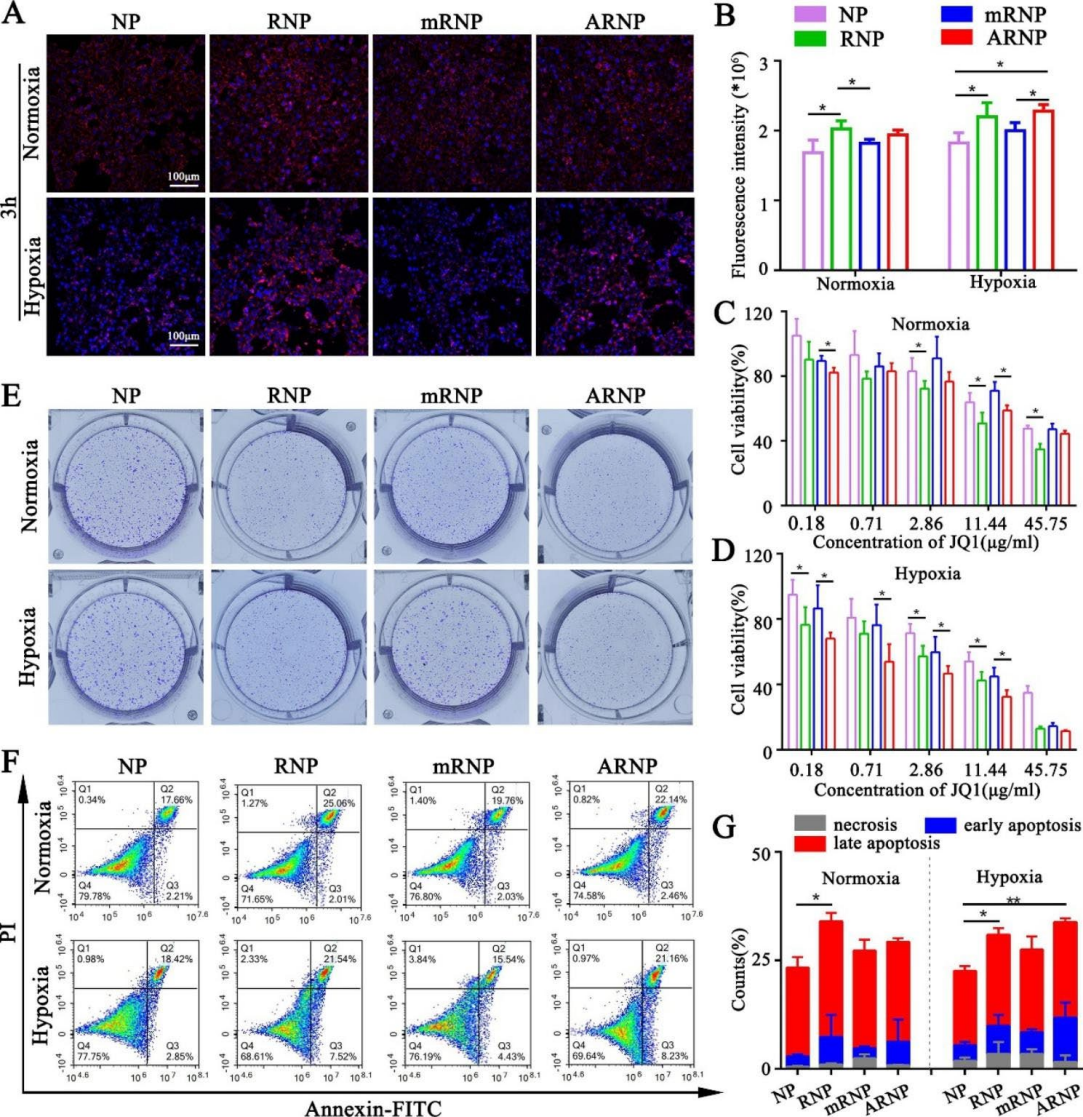
The cellular uptake and antitumor efficacy of the prepared nanoparticles. (**A**) Confocal imaging of cellular uptake under normoxic and hypoxic conditions at 3 h (n = 3). (**B**) Flow cytometry of cellular uptake under normoxic and hypoxic conditions at 3 h (n = 3). (**C**) MTT assay of prepared nanoparticles under normoxic conditions (n = 5). (**D**) MTT assay of prepared nanoparticles under hypoxic conditions (n = 5). (**E**) Colony formation assays of prepared nanoparticles under normoxic or hypoxic conditions (n = 3). (**F**) Flow cytometry assays show apoptosis of prepared nanoparticles under normoxic or hypoxic conditions (n = 3). (**G**) Analysis of apoptosis of prepared nanoparticles under normoxic or hypoxic conditions (n = 3). All data are presented as the mean ± SD. *p < 0.05, **p < 0.01

To investigate the cellular uptake of the prepared nanoparticles in 4T1 cells, the fluorescent probe coumarin 6 (C6) was loaded into the nanoparticles and detected by flow cytometry and confocal imaging. Confocal images showed that C6-RNP had higher cellular uptake than C6-NP under normoxic and hypoxic conditions at 0.5 h (Supplementary Figure S9), indicating that RGD peptides improve cellular uptake. However, C6-mRNP and C6-ARNP showed similar uptake compared with C6-NP (Supplementary Figure S10), which was due to the long PEG chains shielding the RGD peptides. After treatment with C6-loaded nanoparticles for 3 h, confocal images revealed that C6-RNP increased cellular uptake in 4T1 cells compared with C6-NP under normoxic or hypoxic conditions (Fig. 3A), which was attributed to the active targeting of RGD peptides. Notably, C6-ARNP showed higher cellular uptake under hypoxic conditions, but it showed no obvious change under normoxic conditions when compared with C6-mRNP at 3 h (Fig. 3A), suggesting that long PEG chains on the surface of ARNP can be cleaved in response to hypoxia after a certain time, thereby exposing the RGD peptides to facilitate the intracellular uptake of ARNP. The analyses based on flow cytometry were consistent with the confocal imaging results (Fig. 3B), further demonstrating the active targeting role of RGD peptides and the protective role of long cleavable PEG chains.

Next, we evaluated the anticancer efficacy of various prepared nanoparticles under normoxic and hypoxic conditions in vitro. MTT analysis showed that RNP had higher cytotoxicity than NP under both normoxic and hypoxic conditions (Fig. 3C, D), suggesting that RGD peptides enhance the cytotoxicity of nanoparticles. Meanwhile, ARNP showed higher cytotoxicity than mRNP only under hypoxic conditions, demonstrating that ARNP is hypoxia-responsive (Fig. 3C, D). Colony formation assays showed that in comparison with NP, 4T1 cells exhibited a significant reduction in colony formation after treatment with RNP under normoxic or hypoxic conditions for 4 days, indicating that RGD peptides enhance the inhibition of tumor proliferation. Moreover, ARNP effectively inhibited colony formation under normoxic and hypoxic conditions when compared with mRNP (Fig. 3E). These results suggested that ARNP can also respond to azoreductases produced by 4T1 cells under hypoxic conditions over time, resulting in long PEG chain cleavage and inhibition of cell proliferation. In addition, flow cytometry of apoptosis showed that the early apoptosis and late apoptosis of 4T1 cells treated with RNP were higher than those of NP under normoxic and hypoxic conditions, but treatment with ARNP resulted in relatively high levels of apoptosis compared with mRNP under hypoxic conditions, even though there were no significant differences (Fig. 3F, G). Collectively, these data demonstrated that ARNP has effective cellular uptake and antitumor properties under hypoxic conditions in vitro.

### ARNP targets both primary tumor and bone metastasis efficiently in vivo

**Fig. 4:**
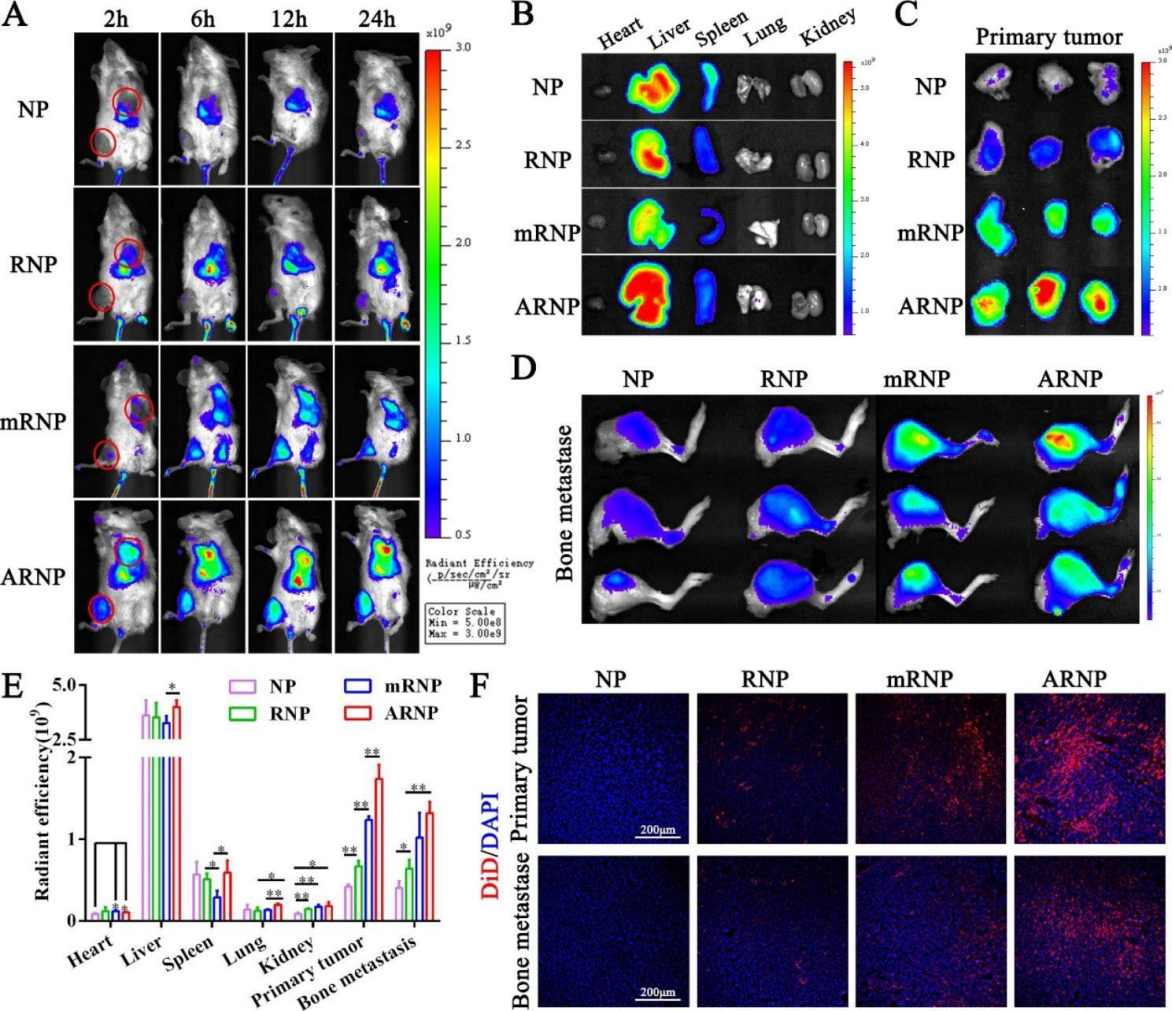
ARNP efficiently accumulates in primary tumors and bone metastases. (**A**) In vivo DiD fluorescence images showing the primary tumor and bone metastasis of different intravenous treatments at different time points. (**B**) Ex vivo images showing the biodistribution of NP, RNP, mRNP and ARNP in major organs (left to right: heart, liver, spleen, lung, kidney) at 24 h post-injection. (**C**) Ex vivo fluorescence images of primary tumors at 24 h postinjection. (**D**) Ex vivo fluorescence images of bone metastasis at 24 h post-injection. (**E**) Semiquantitative analysis of major organs and tumors based on ex vivo fluorescence images. (**F**) Confocal images showing the distribution of NP, RNP, mRNP and ARNP in primary tumors and bone metastases (blue represents DAPI, red represents DiD). All data are presented as the mean ± SD (n = 3). *p < 0.05, **p < 0.01

To study the biodistribution of nanoparticles we prepared, a fluorescent probe, DiD, was encapsulated in nanoparticles and then detected by an in vivo imaging system. As shown in Fig. 4A, RNP moderately increased the DiD fluorescence distribution in primary tumors and bone metastases compared with NP, which was due to the active targeting of RGD peptides. Unexpectedly, when the nanoparticles were modified with longer PEG chains, mRNP and ARNP significantly accumulated in primary tumors and bone metastases compared with NP and RNP, indicating the enhanced EPR effect. Importantly, ARNP was distributed more effectively in tumors than mRNP at all time points, suggesting that long PEG chains can be broken in tumors and subsequently promote their intratumoral enrichment through RGD-αvβ3 integrin interactions. The ex vivo fluorescence images demonstrated that all four nanoparticles mainly accumulated in the liver and spleen at 24 h after intravenous injection (Fig. 4B). The ex vivo images of the primary tumor were consistent with those of the in vivo imaging at 24 h and showed that ARNP had the highest tumor accumulation compared with the other groups (Fig. 4C). The results of ex vivo images also demonstrated that ARNP displayed the highest distribution in bone metastasis among the four nanoparticles (Fig. 4D). Semi-quantification of ex vivo imaging showed that ARNP significantly accumulated 1.41-fold, 2.58-fold and 4.11-fold more in primary tumors than mRNP, RNP and NP, respectively (Fig. 4E). In addition, the semiquantitative results also showed that ARNP had a higher biodistribution in bone metastasis than RNP (Fig. 4E), suggesting that RGD peptides shielded by long cleavable PEG chains improve the targeting enrichment of nanoparticles in bone metastasis. Next, we applied confocal imaging to study the biodistribution of nanoparticles in tumor tissue. According to confocal imaging, the DiD fluorescence distribution of ARNP in both primary tumors and bone metastases was the highest among the nanoparticles we developed (Fig. 4F), which was consistent with the results of in vivo and ex vivo imaging studies. These results jointly validated that ARNP can efficiently accumulate in primary tumors and bone metastases through the enhanced EPR effect and active targeting of RGD peptides, thereby ameliorating the limitation of advanced uptake by normal cells caused by direct exposure to RGD peptides.

### ARNP inhibits the growth of primary tumors in mice

**Fig. 5:**
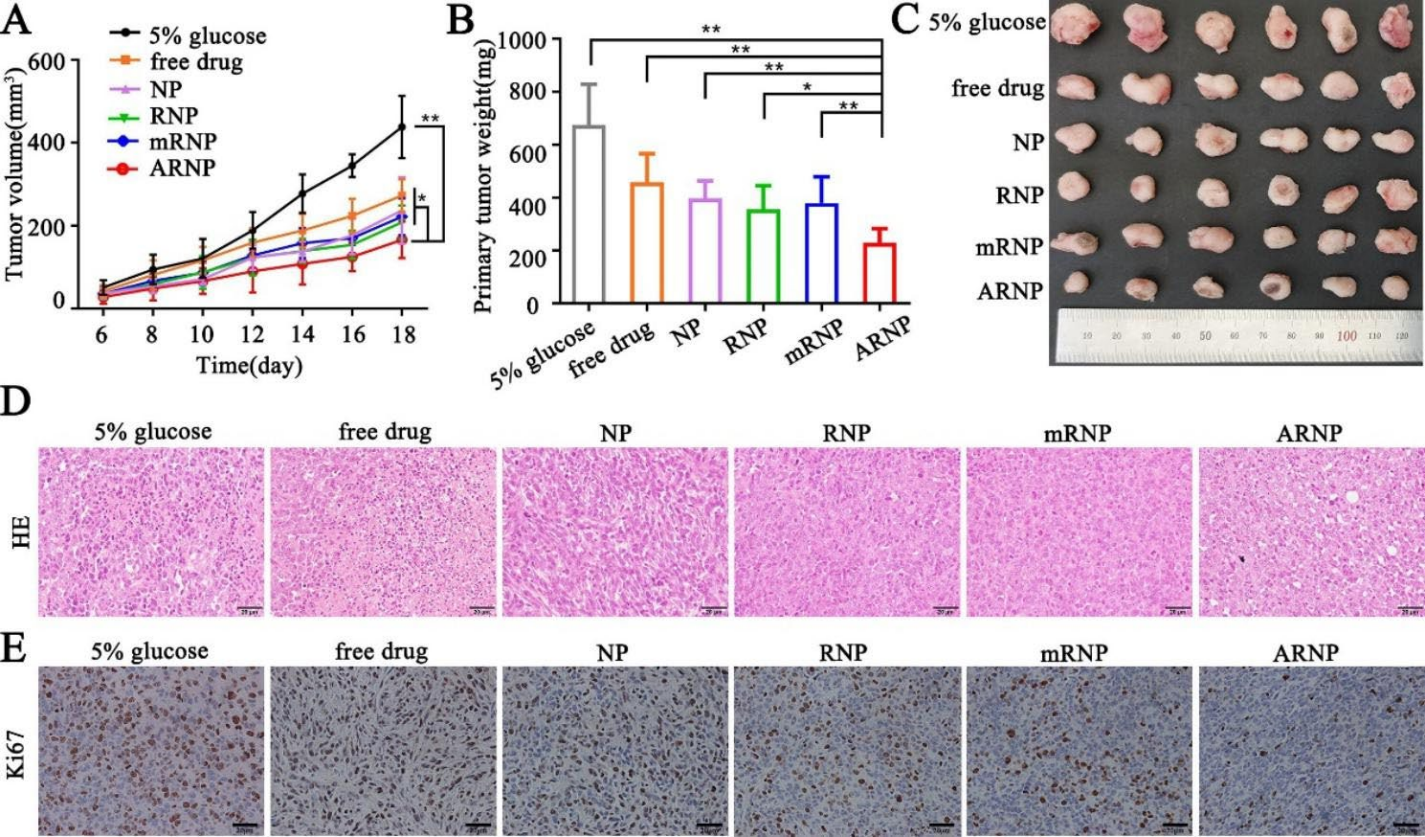
ARNP inhibits the growth of primary tumors in vivo. (**A**) Tumor volume of the primary tumor recorded every two days in mice. (**B**) Tumor weights and (**C**) tumor images of primary tumors in mice administered different treatments. (**D**) Representative images of H&E staining and (**E**) Ki67 immunohistochemical staining of primary tumors from different groups (scale bar represents 20 μm). All data are presented as the mean ± SD (n = 6). *p < 0.05, **p < 0.01

The above results revealed that ARNP exhibited promising cytotoxicity and tumor distribution; therefore, we further evaluated the antitumor effect of ARNP in vivo. Compared with the rapid tumor growth curve of the 5% glucose group, groups treated with different drugs displayed relatively slower growth rates. Notably, the ARNP group showed the smoothest growth curve (Fig. 5A), indicating the better antitumor effect of ARNP treatment. The tumor weights (Fig. 5B) and images of tumors under natural light on Day 18 (Fig. 5C) were consistent with the measurements of tumor volume in vivo. The tumor weight data showed that all drug treatments suppressed primary tumor growth compared with the 5% glucose group. Most importantly, ARNP suppressed 65.8% of tumor growth compared with the 5% glucose group, which was 1.5-fold, 1.39-fold, 1.61-fold and 2.0-fold higher than that of the mRNP, RNP, NP and free drug groups, respectively. These results jointly confirmed that ARNP can achieve the best antitumor effect by combining the enhanced EPR effect and active targeting. Hematoxylin and eosin (H&E) staining showed that in comparison with 5% glucose, treatment with drug-loaded nanoparticles markedly increased nuclear damage and degradation, especially ARNP treatment (Fig. 5D). Ki67 protein is an important marker of cell proliferation and is involved in the development of breast cancer. Immunohistochemical staining revealed that treatment with ARNP significantly decreased the expression of Ki67 compared to other treatments (Fig. 5E), indicating the inhibition of cell proliferation. In addition, the body weights of all the groups showed no significant change during the treatment process (Supplementary Figure S5). Images of H&E staining showed no obvious histological changes in the major organs of any of the groups (Supplementary Figure S11). Moreover, there were no significant differences in hematological indicators, including while blood cell counts and the serum levels of ALT, AST, CREA, UREA and LDH (Supplementary Figure S12). These results indicated no apparent toxicity of any of the nanoparticles we prepared. In summary, we suggested that ARNP improves antitumor effects with low toxicity and side effects, largely due to the enhanced accumulation and drug synergistic effect.

### Constructed nanoparticles inhibit the proliferation of bone metastasis and alleviate osteolysis

**Fig. 6:**
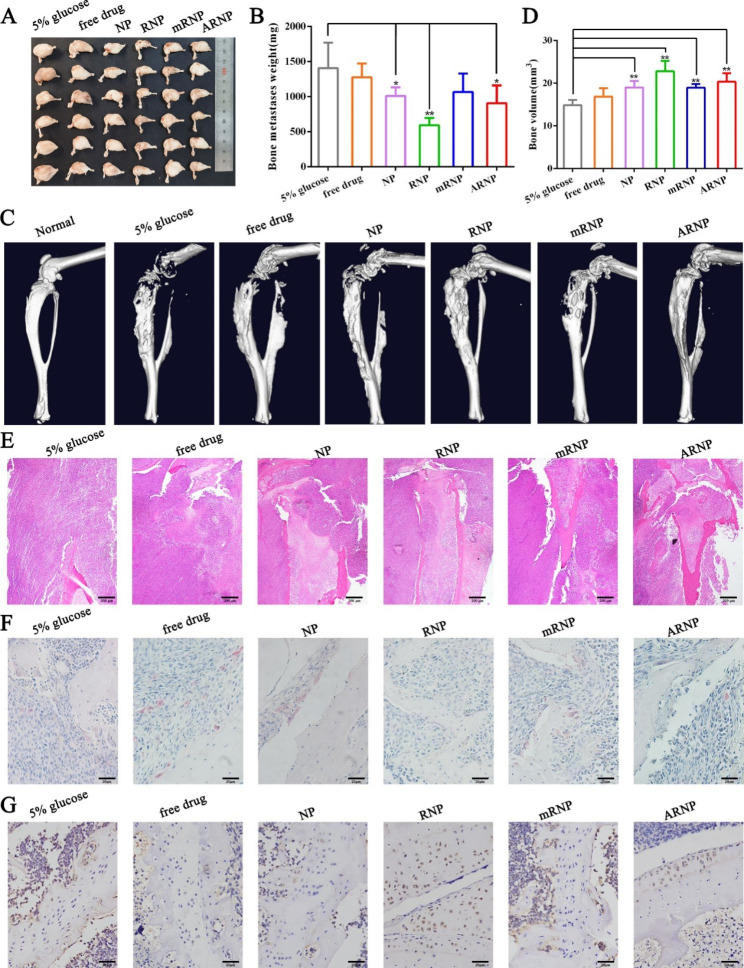
The constructed nanoparticles inhibited the proliferation of bone metastasis and alleviated osteolysis. (**A**) Photographic image of bone-metastatic tumors from different treatment groups. (**B**) Bone-metastatic tumor weights of different treatment groups. (**C**) Micro-CT reconstruction images of the tumor-bearing limbs from different groups. (**D**) Bone volume analyses of different treatment groups. (**E**) H&E staining, (**F**) TRAP staining and (**G**) OCN immunohistochemical staining images of the tumor-bearing limbs from different groups. All data are presented as the mean ± SD (n = 6). *p < 0.05, **p < 0.01

Considering the adverse effects of bone metastasis on the survival of breast cancer patients, we further explored the therapeutic effect of nanoparticles on bone metastasis. After carefully dissecting the tumor-bearing limb muscles, the image of bone metastasis under natural light on Day 18 revealed that RNP treatment displayed a remarkable antimetastatic effect compared with the other four groups, while NP and ARNP treatment showed a moderate inhibitory effect compared to the 5% glucose group (Fig. 6A). The bone-metastatic tumor weights were consistent with the natural light image. The average weight of bone-metastatic tumors in the NP, RNP and ARNP groups was 1.4-fold, 2.4-fold and 1.6-fold lower than that of the 5% glucose group, respectively (Fig. 6B), indicating the bone metastasis inhibitory effect of nanoparticles. Next, microcomputed tomography (micro-CT) and histological assays were applied to investigate bone destruction in tumor-bearing limbs. Micro-CT demonstrated that in contrast with the smooth bone surface in tibias and femurs from normal hind limbs, tumor-bearing limbs from the 5% glucose group displayed rough bone surfaces and severe bone erosion, indicating bone destruction of bone metastasis. However, when treated with drug-loaded nanoparticles, bone erosion was remarkably reduced compared with that in the 5% glucose group (Fig. 6C). Similarly, the total bone volume analyses showed that the bone density was increased after treatment with drug-loaded nanoparticles, reflecting the effective bone protection of nanoparticles we developed (Fig. 6D). The extent of damage to the hind limb with metastatic tumors was further investigated by H&E staining. The bone trabeculae and bone marrow cavity in the 5% glucose and free drug groups were invaded by proliferating tumor cells. In contrast, bone osteolysis in the drug-loaded nanoparticle groups was suppressed, with a relatively complete bone structure (Fig. 6E). Osteoclasts are responsible for bone resorption and participate in the development of breast cancer [37]. TRAP staining of bone slices demonstrated that the 5% glucose and free drug groups had a large number of osteoclasts, while there were reduced TRAP^+^ osteoclasts in the drug-loaded nanoparticle groups, especially the RNP and ARNP groups (Fig. 6F), indicating the osteoclast inhibition of the nanoparticles we prepared. To study bone remodeling, the osteoblast marker OCN was detected by immunohistochemical staining. The results showed that the expression of OCN in bone tissue of the RNP and ARNP groups markedly increased compared with that in the 5% glucose and free drug groups (Fig. 6G), suggesting enhanced bone remodeling after treatment with RNP and ARNP. Thus, these data revealed that the nanoparticles we developed, especially RNP and ARNP, can not only inhibit the proliferation of bone metastatic tumor cells but also suppress the osteolysis of hind limbs with metastatic tumors.

### ARNP suppresses pulmonary metastasis secondary to bone metastasis

**Fig. 7:**
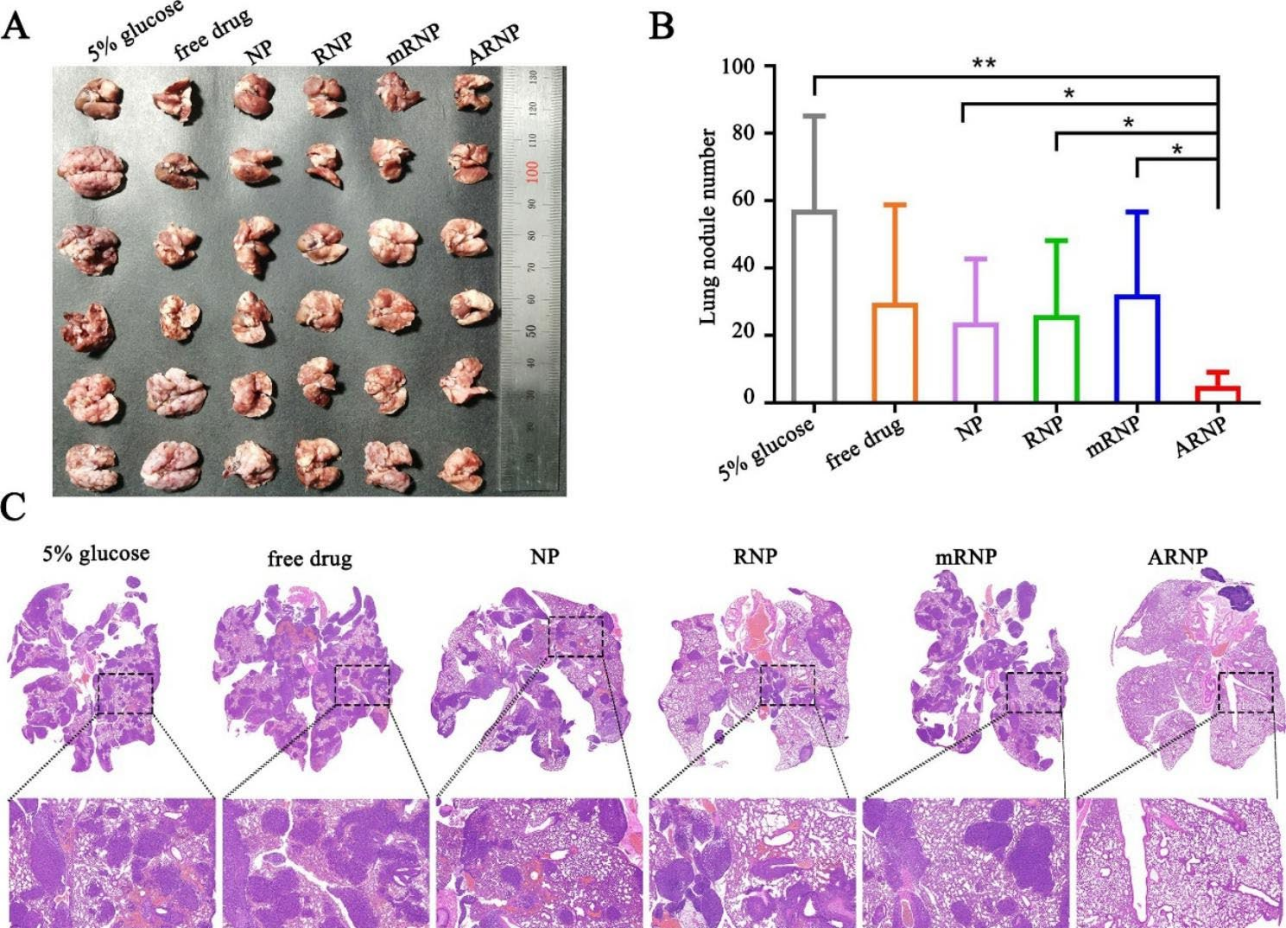
ARNP suppresses pulmonary metastases secondary to bone metastasis. (**A**) Photographic image of lung secondary metastases from different treatment groups. (**B**) Lung metastatic nodule numbers in the different treatment groups. (**C**) Lung H&E staining of different treatment groups. All data are presented as the mean ± SD (n = 6). *p < 0.05, **p < 0.01

Additionally, when we harvested the mouse organs, we unexpectedly found secondary metastatic pulmonary nodules. As shown in Fig. 7A, the 5% glucose group showed obvious lung metastases with significant nodules. NP treatment resulted in moderate inhibition of lung secondary metastases compared with the 5% glucose group, while the ARNP group showed significant suppression of lung secondary metastases when compared with the control group. The analysis of lung nodule number revealed that ARNP treatment suppressed 91.3% of lung metastases compared with the 5% glucose group even though individual differences existed (Fig. 7B), indicating that ARNP effectively inhibits secondary lung metastases. H&E staining of lung slices was consistent with the lung nodule analysis results. Obvious lung tumor nodules were observed in the 5% glucose and free drug groups, while the ARNP group showed negligible metastatic nodules, which was similar to normal lungs (Fig. 7C). Previous studies have revealed that breast cancer cells can be educated to obtain further secondary metastatic ability after metastasis to bone, exacerbating the poor clinical outcome [38]. Our results also suggested that bone metastasis promoted spontaneous metastasis to the lung. Due to the severe effect of secondary metastasis, it is important to prevent or inhibit the metastasis of cancer cells from bone. ARNP significantly inhibited lung metastasis secondary to bone metastasis, showing promising potential in breast cancer treatment. The suppression mechanism of ARNP may be due to enhanced tumor site accumulation and high cellular uptake, leading to a more effective tumor-inhibiting ability.

## Conclusion

In summary, we suggested that JQ1 and icaritin had an effective synergistic role in anti-breast cancer cells, which could help to reduce the drug resistance of JQ1. To effectively deliver JQ1 and icaritin to the tumor site, we developed a hypoxia-cleavable, RGD peptide-modified PLGA nanoparticle (termed ARNP). The decoration of long cleavable PEG chains could shield RGD peptides, thereby extending the circulation time of nanoparticles and reducing cellular uptake at nonspecific sites in vivo. Under a hypoxic tumor microenvironment, ARNP showed efficient cellular uptake in breast cancer cells via RGD-αvβ3 integrin interaction after cleavage of long PEG chains. In vivo and in vitro experiments strongly demonstrated that ARNP had enhanced cytotoxicity, good biodistribution and bone remodeling effects, thereby treating breast cancer and bone metastasis. In short, the nanomedicine ARNP with hypoxia-responsive cleavage and active targeting provided a new simple but practical paradigm for epigenetic drug delivery to treat breast cancer and bone metastasis.

## Materials and methods

### Materials

JQ1 (Catalog# J844079) was obtained from Macklin (Shanghai, China), and icaritin (Catalog# 118525-40-9) was obtained from Energy Chemical (Shanghai, China). The chemical structures of JQ1 and icaritin are shown in Supplementary Figure S1. Poly(D,L-lactide-co-glycolide 50/50) (PLGA21000) was obtained from Jinan Daigang Biomaterial company (Jinan, China). 1,2-Distearoyl-sn-glycero-3-phosphoethanolamine-N-[methoxy(polyethyleneglycol)-2000] (DSPE-mPEG2000), DSPE-PEG2000-maleimide, and DSPE-mPEG5000 were purchased from Ponsure Biotechnology (Shanghai, China). Hypoxic-responsive DSPE-azobenzene-mPEG5000 (DSPE-Azo-mPEG5000) was custom-synthesized by Ruixibio Ltd. (Xi’an, China). Cys-RGD peptide was purchased from Sangon Biotech (Shanghai, China). 1,1′-dioctadecyl-3,3,3′,3′-tetramethyl indodicarbocyanine, 4-chlorobenzenesulfonatesalt (DiD) (Catalog # M9379) was purchased from ChemBridge (San Diego, USA). An Annexin V-FITC/PI Apoptosis Detection Kit (Catalog# 40,302) was obtained from Yeasen Biotechnology Co., Ltd. (Shanghai, China).

### Synthesis and characterization of DSPE-PEG chains

DSPE-PEG2000-RGD was synthesized via the maleimide-thiol coupling reaction. DSPE-PEG2000-maleimide and Cys-RGD (molar ratio = 1:1.2) were reacted in a solvent mixture comprising phosphate buffered saline (pH = 7.5) with gentle stirring at room temperature and without oxygen for 24 h. Then, DSPE-PEG2000-RGD was purified by dialysis (MWCO1000) with ultrapure water for 48 h, and the solution was collected and lyophilized. The successful synthesis of DSPE-PEG2000-RGD was characterized by ^1^ H-NMR spectroscopy and MALDI-TOF-MS.

DSPE-Azo-mPEG5000 and DSPE-mPEG5000 were dissolved in ultrapure water at a concentration of 1.6 mg/ml. Subsequently, Na_2_S_2_O_4_ was added into above solution to a final concentration of 5mM and sealed in a quartz cuvette for 16 h. The hypoxia-responsive property of DSPE-Azo-mPEG5000 was detected by the UV-Vis spectrophotometer.

### Preparation and characterization of NP, RNP, mRNP and ARNP

The preparation of drug-loaded NP was modified on the basis of the emulsion/solvent evaporation method. Briefly, PLGA (21,000, 50/50), JQ1 and icaritin (m/m, JQ1: icaritin = 2:1) were dissolved in acetonitrile to form the oil phase. DSPE-mPEG2000 was dissolved in ultrapure water to form the aqueous phase. Subsequently, the mixed oil phase was added to the aqueous phase (v/v, oil phase: aqueous phase = 1:10). The mixture was vortexed for 10 s, and then the acetonitrile was evaporated at low pressure. Finally, the mixed solution was centrifuged to remove unencapsulated drugs to obtain purified drug-loaded NP. Drug-loaded RNP was prepared as indicated above. PLGA (21,000, 50/50), JQ1 and icaritin (m/m, JQ1: icaritin = 2:1) were dissolved in acetonitrile, and DSPE-PEG2000-RGD was dissolved in ultrapure water to form the aqueous phase. To obtain drug-loaded mRNP, PLGA (21,000, 50/50), JQ1 and icaritin (m/m, JQ1: icaritin = 2:1) were dissolved in acetonitrile, and DSPE-PEG2000-RGD and DSPE-mPEG5000 were dissolved in ultrapure water to form the aqueous phase. For drug-loaded ARNP, PLGA (21,000, 50/50), JQ1 and icaritin (m/m, JQ1: icaritin = 2:1) were dissolved in acetonitrile, and DSPE-PEG2000-RGD and DSPE-Azo-mPEG5000 were dissolved in ultrapure water to form the aqueous phase. The mass ratio of PLGA and DSPE-PEG chains (including DSPE-mPEG2000, DSPE-PEG2000-RGD, DSPE-mPEG5000 and DSPE-Azo-mPEG5000) was 10:1.

The particle sizes and zeta potentials of NP, RNP, mRNP and ARNP were determined by dynamic light scattering (DLS, Brookhaven) and measured by transmission electronic microscopy (TEM, H-600, Hitachi, Japan). The encapsulation efficiency (EE%) and drug loading efficiency (DL%) were determined by ultraviolet spectrophotometry. The prepared nanoparticles were incubated in medium containing 10% FBS at 37 °C for 24 h, and the size changes were determined by DLS to detect the stability.

#### Cellular uptake study

4T1 cells were seeded in 12-well plates and cultured for 24 h under normoxic conditions (21% O_2_). Afterward, the cells were treated with C6-NP, C6-RNP, C6-mRNP and C6-ARNP (at the same C6 concentration of 100 ng/mL) in serum-free medium. The cells were then divided into two groups. One group was cultured under normoxic conditions, and the other was cultured under hypoxic conditions (2% O_2_) using a hypoxia incubator (MIC101, Billups-Rothenberg). After incubating for 0.5 or 3 h, the cells were collected, and the fluorescence intensity of C6 was detected by flow cytometry (BD FACSCelesta, USA). For the qualitative analysis, cells seeded in glass-bottomed dishes were treated as above. Then, the cells were fixed and stained with DAPI for 5 min. Fluorescence images were obtained by confocal microscopy.

#### Apoptosis study

For the free drug, 4T1 cells were treated with JQ1, icaritin or both (the equivalent of 5.4 µg/mL JQ1 and 2.7 µg/mL icaritin) for 6 h. For drug-loaded nanoparticles, 4T1 cells were treated with NP, RNP, mRNP and ARNP (the equivalent of 5.4 µg/mL JQ1 and 2.7 µg/mL icaritin). The nanoparticle-treated cells were then divided into two groups and cultured under normoxic and hypoxic conditions for 6 h. After incubation, the cells were collected and stained with Annexin V-FITC and propidium iodide according to the manufacturer’s instructions and measured by flow cytometry.

#### Cytotoxicity

In vitro cytotoxicity was measured by MTT assay. 4T1 cells seeded in 96-well plates (5000 cells per well) were cultured for 24 h. The culture medium was replaced with different formulations at different concentrations, and the cells were cultured under normoxic or hypoxic conditions for another 24 h. The cells were then incubated with MTT reagent (5 mg/mL, 10 µL) for 4 h. Formazan crystals were dissolved in 150 µL DMSO, and the absorbance at 570 nm was detected using a microplate reader (Thermo Scientific Varioskan Flash). The CI was calculated by the Chou-Talalaly method using CalcuSyn software. CI values < 0.3, 0.3–0.9 and > 1.1 indicate strong synergism, synergism and antagonism, respectively.

#### Colony formation assay

4T1 cells (2000 cells per well) were seeded in 6-well plates and cultured for 24 h under normoxic conditions. For the free drug, 4T1 cells were treated with JQ1, icaritin or both (the equivalent of 2.6 µg/mL JQ1 and 1.3 µg/mL icaritin) for 4 days. For drug-loaded nanoparticles, 4T1 cells were treated with NP, RNP, mRNP and ARNP (the equivalent of 2.6 µg/mL of JQ1 and 1.3 µg/mL of icaritin). The nanoparticle-treated cells were then divided into two groups and cultured under normoxic and hypoxic conditions for 4 days. After fixation with 4% paraformaldehyde, the cells were stained with 0.1% crystal violet (Beyotime) and photographed using a camera.

#### Western blotting

Western blotting was performed according to our previous study [39]. 4T1 cells were treated with JQ1, icaritin or both (the equivalent of 5.4 µg/mL JQ1 and 2.7 µg/mL icaritin) for 12 h. The cells were then lysed in RIPA solution containing phosphatase inhibitor and protease inhibitor. The protein concentration was measured by the BCA method, and equal amounts of each sample were diluted in 5× sodium dodecyl sulfate (SDS) loading buffer and denatured by boiling. Protein lysates were separated by SDS polyacrylamide gel electrophoresis and transferred to polyvinylidene difluoride membranes (Millipore). The membranes were blocked in 5% nonfat dry milk dissolved in TBST at room temperature for 1 h and then incubated overnight with primary antibodies against c-myc (1:2000, 10828-1-AP, Proteintech) and GAPDH (1:3000, AF0006, Beyotime) overnight at 4 °C. The protein signals were detected using chemiluminescent reagents (Millipore) according to the manufacturer’s instructions.

#### Primary tumor and bone metastasis model

All animal experiments were performed under the guidelines, evaluated and approved by the ethics committee of Sichuan University. The tumor model was developed according to our previous study [40]. 4T1 cells (2 × 10^5^) were injected subcutaneously into the third left mammary fat pads, and 1 × 10^5^ 4T1 cells were injected into the tibia of female BALB/c mice to construct primary tumor and bone metastasis models. Mice with similar size primary tumors and bone metastatic tumors were selected and randomly divided into different groups to conduct follow-up experiments.

#### Tissue biodistribution

Tumor models were established and intravenously injected with DiD-NP, DiD-RNP, DiD-mRNP and DiD-ARNP via the tail vein. The biodistribution of DiD in the primary tumor and bone metastatic tumor was analyzed at 2, 6, 12 and 24 h after administration by applying the Lumina III Imaging System (PerkinElmer, USA). At the end of this experiment, all the mice were sacrificed. Major organs (heart, liver, spleen, lung, and kidney) and tumors were collected for ex vivo imaging of DiD fluorescence.

#### In vivo antitumor effect

Breast cancer models were constructed, and mice bearing primary tumors of approximately 50 mm^3^ were randomized into 6 groups. The groups were intravenously administered 5% glucose, free drug, NP, RNP, mRNP or ARNP at an equivalent dose (15 mg kg^− 1^ for JQ1 and 7.5 mg kg^− 1^ for icaritin). The mice were treated with various formulations every second day. Tumor volume and body weight were recorded every other day during the treatment. All mice were sacrificed on the 18th day after tumor implantation. Primary tumors and bone metastatic tumors were collected, weighed and captured. Major organs (heart, liver, spleen and kidney) were collected, and slides were made for H&E staining to evaluate the biosafety of the nanoparticles. The lungs were captured, and the pulmonary nodules were calculated according to our previous study [41]. The pulmonary nodules were divided into four grades: grade I < 0.5 mm; 0.5 mm ≤ grade II < 1 mm; 1 mm ≤ grade III < 2 mm; grade IV > 2 mm. Pulmonary nodule numbers were calculated as I×1 + II×2 + III×3 + IV×4.

#### Micro-CT analysis and bone staining

Tumor-bearing limbs were fixed in 4% paraformaldehyde and scanned at 90 kV and 88 µA with a voxel size of 72 μm by microcomputed tomography (Micro-CT, PerkinElmer, Quantum GX II, USA). Bone volume was calculated based on the same anatomical origin and end point. After analysis by micro-CT, the tumor-bearing limbs were transferred to ethylenediaminetetraacetic acid-glycerol solution for decalcification. The decalcified limbs were subsequently embedded in paraffin and then sectioned for H&E, OCN and TRAP staining.

#### Safety assessment

Tumor models were sacrificed, and the blood and major organs (heart, liver, spleen and kidney) were collected for serum enzyme and H&E staining analyses.

#### Statistical analysis

All data are presented as the mean ± standard deviation (SD). Student’s two-sided t test and one-way analysis of variance (ANOVA) were performed for two-group comparisons and multiple group comparisons, respectively. Statistical significance was set at *p < 0.05, **p < 0.01 and ***p < 0.001.

## Electronic supplementary material

Below is the link to the electronic supplementary material.

Supplementary Material 1

## Data Availability

The data that support the findings of this study are available from the corresponding author upon reasonable request.
