# Supplementary material for: Hypoxia-cleavable and specific targeted nanomedicine delivers epigenetic drugs for enhanced treatment of breast cancer and bone metastasis
Source: J Nanobiotechnology. 2023 Jul 12;21:221. doi: 10.1186/s12951-023-01939-7 (PMC10337106; doi:10.1186/s12951-023-01939-7)
Supplement: Supplementary file 1 — Supplementary Material 1 [file 12951_2023_1939_MOESM1_ESM.docx]

**Hypoxia-cleavable and specific targeted nanomedicine delivers epigenetic drugs for enhanced treatment of breast cancer and bone metastasis**

Zhaofeng Li^a,b,1^, Peixin Liu^a,1^, Wei Chen^d,1^, Xueying Liu^a^, Fan Tong^b^, Junhui Sun^a^, Yang Zhou^b^, Ting Lei^b^, , Wenqin Yang^b^, Dong Ma^c,*^, Huile Gao^b,*^, Yi Qin^a,*^

**Supplementary Information**


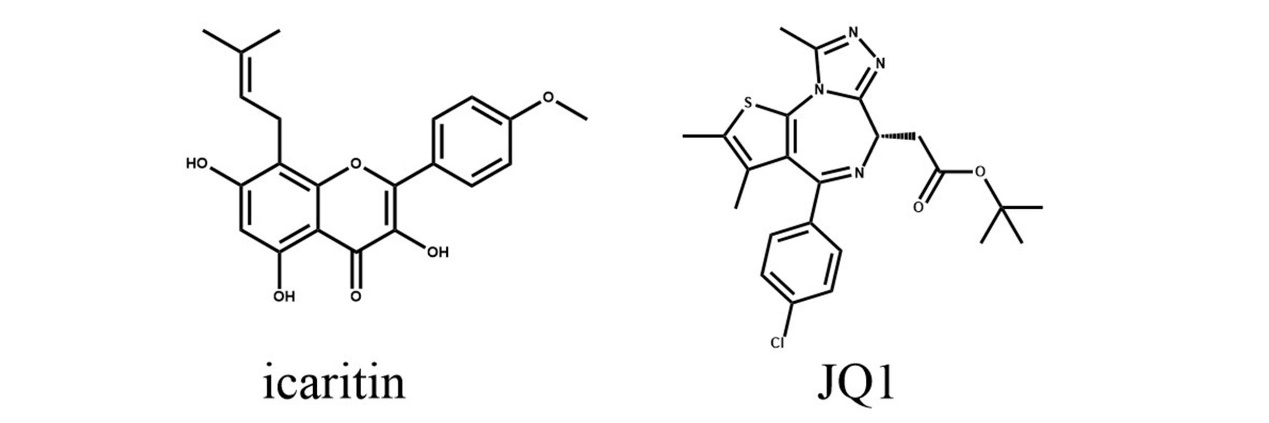


**Supplementary Figure 1.** The chemical structures of JQ1 and icaritin.


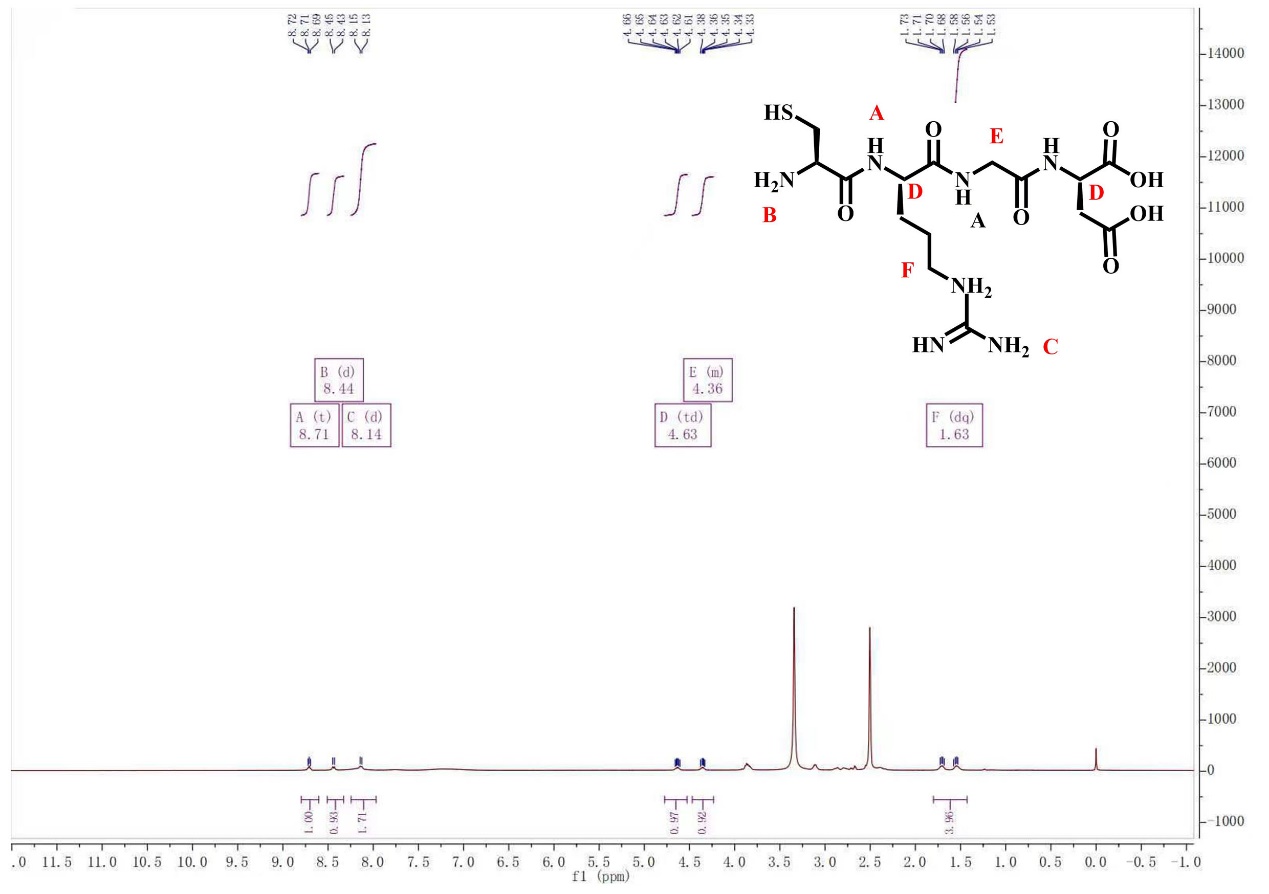


**Supplementary Figure 2.** ^1^HNMR spectra (400 MHz) of CRGD.


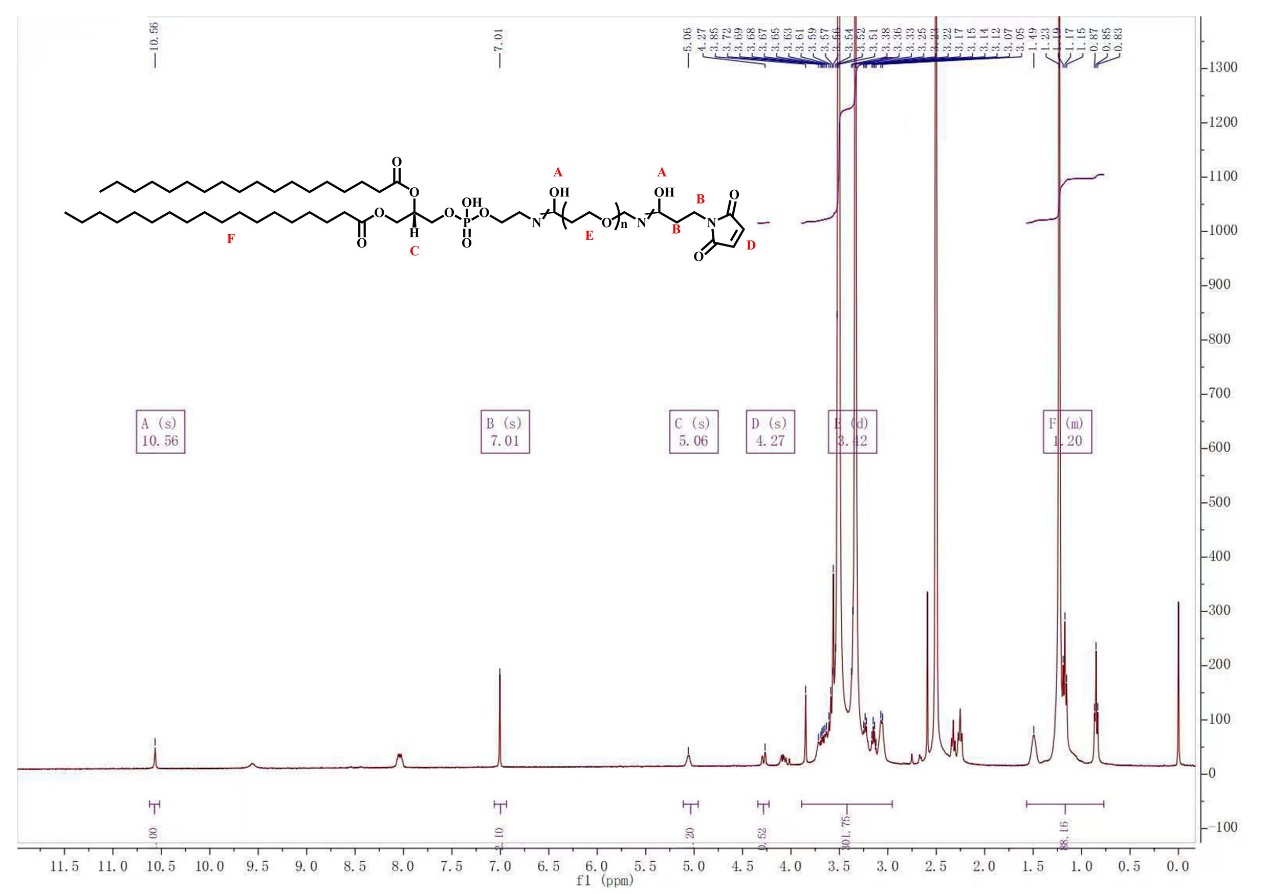


**Supplementary Figure 3.** ^1^HNMR spectra (400 MHz) of DSPE-PEG2000-Mal.


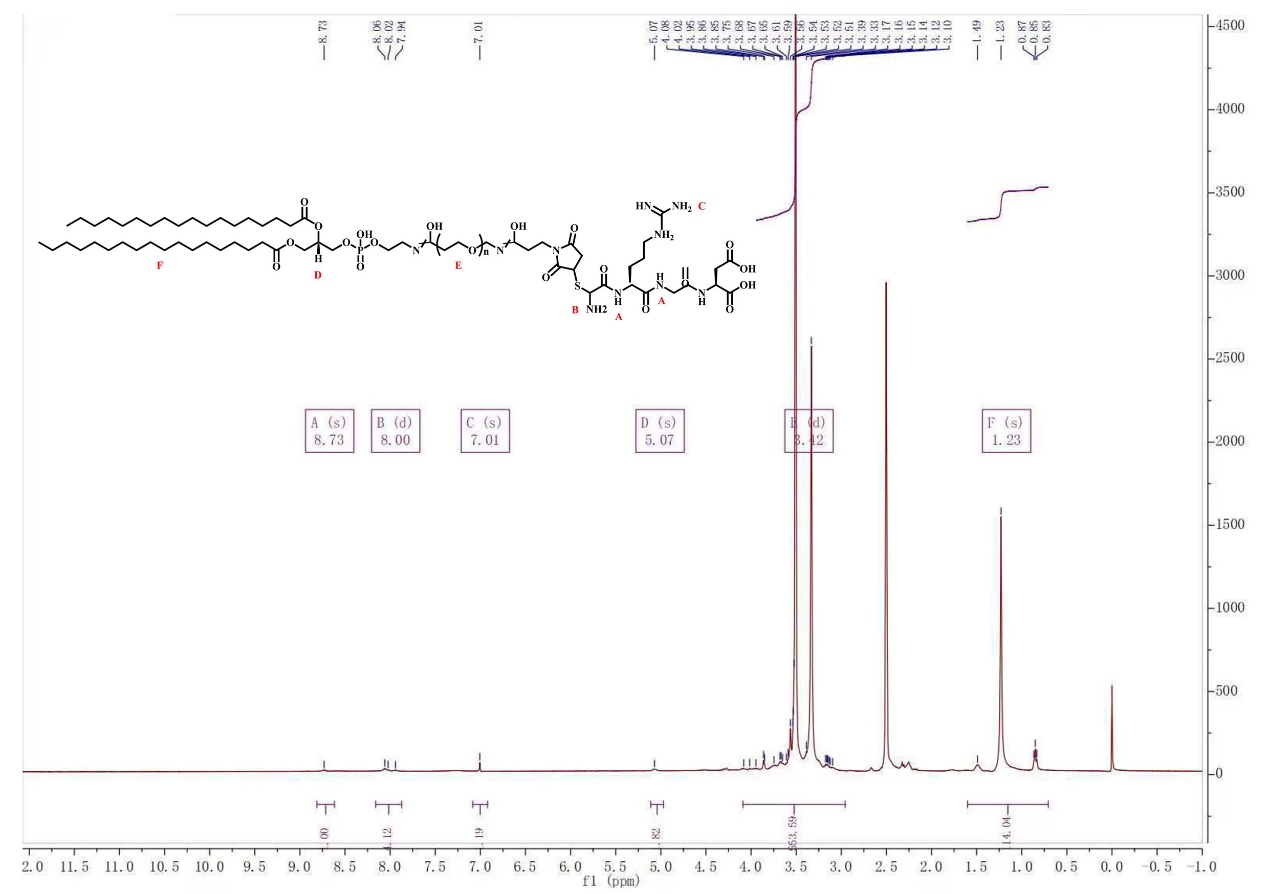


**Supplementary Figure 4.** ^1^HNMR spectra (400 MHz) of DSPE-PEG2000-RGD.


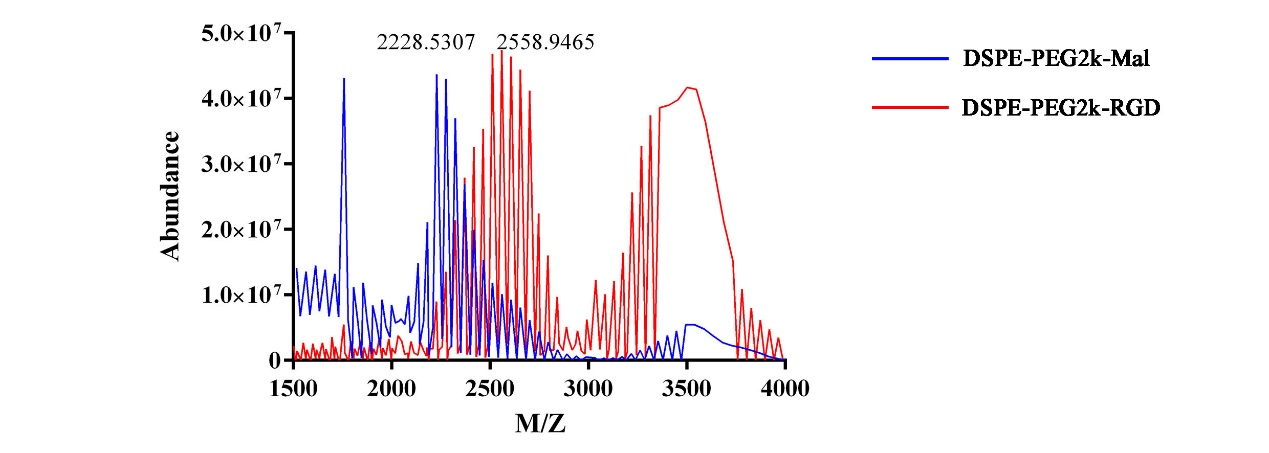
**Supplementary Figure 5.** MALDI-TOF-MS analyses of DSPE-PEG2000-Mal and DSPE-PEG2000-RGD.


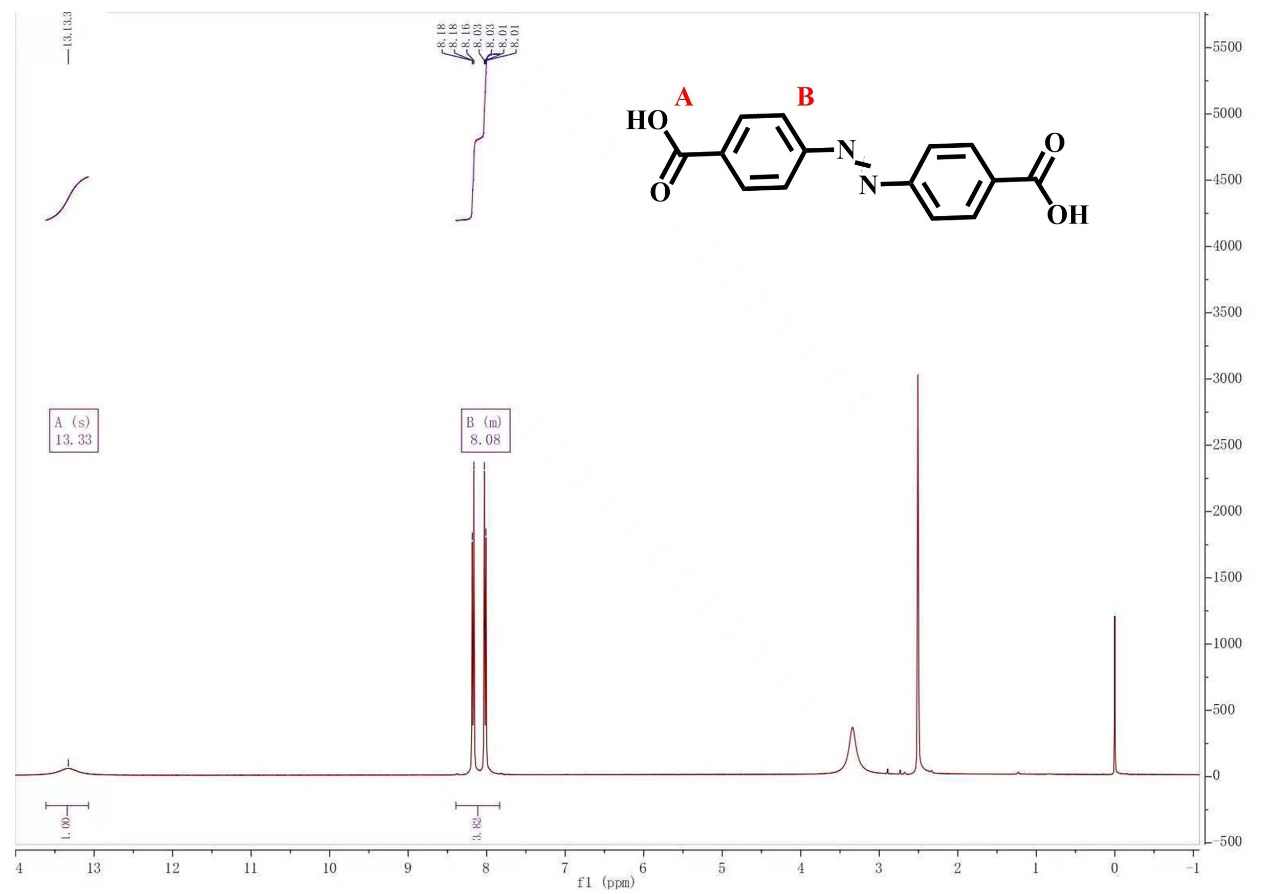
**Supplementary Figure 6.** ^1^HNMR spectra (400 MHz) of azobenzene.


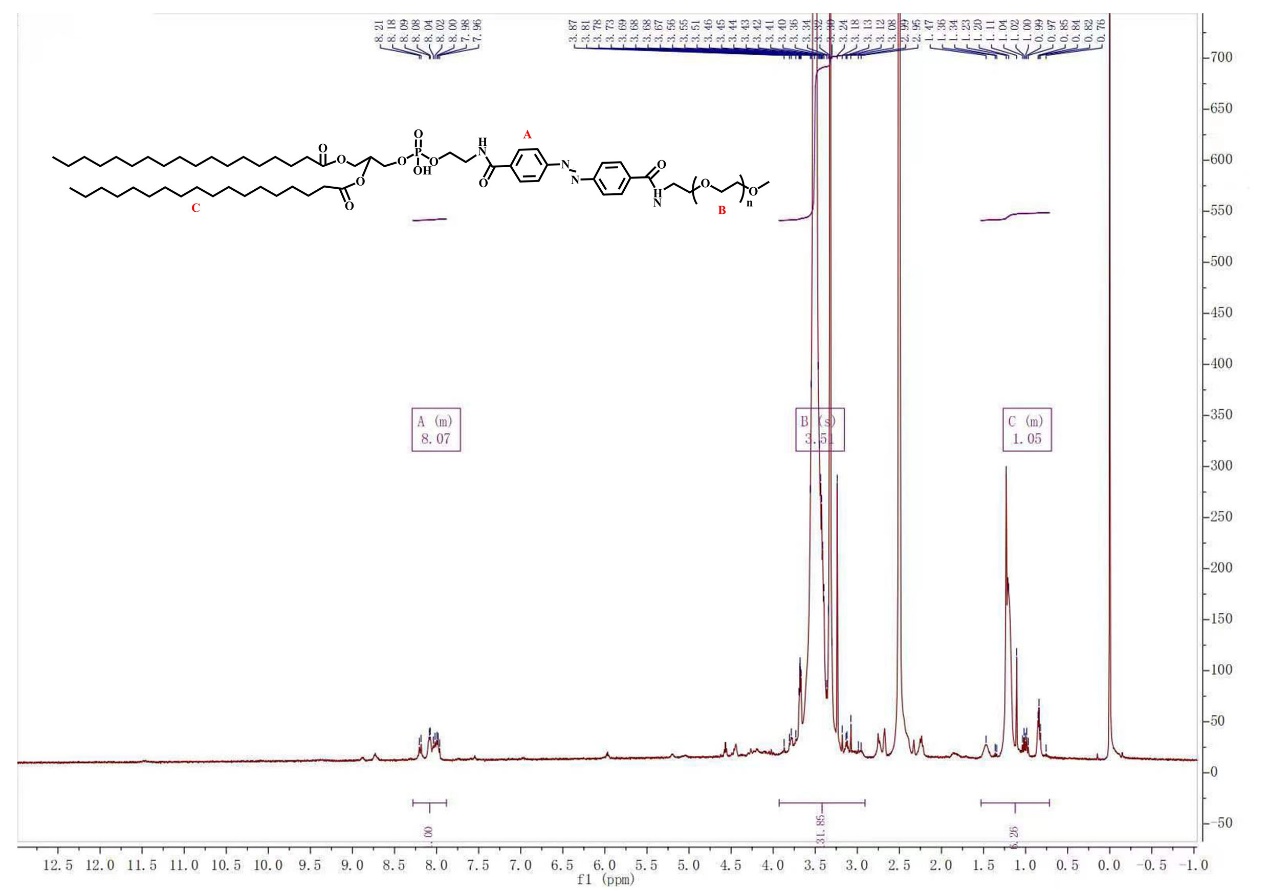
**Supplementary Figure 7.** ^1^HNMR spectra (400 MHz) of DSPE-Azo-mPEG5000.

**
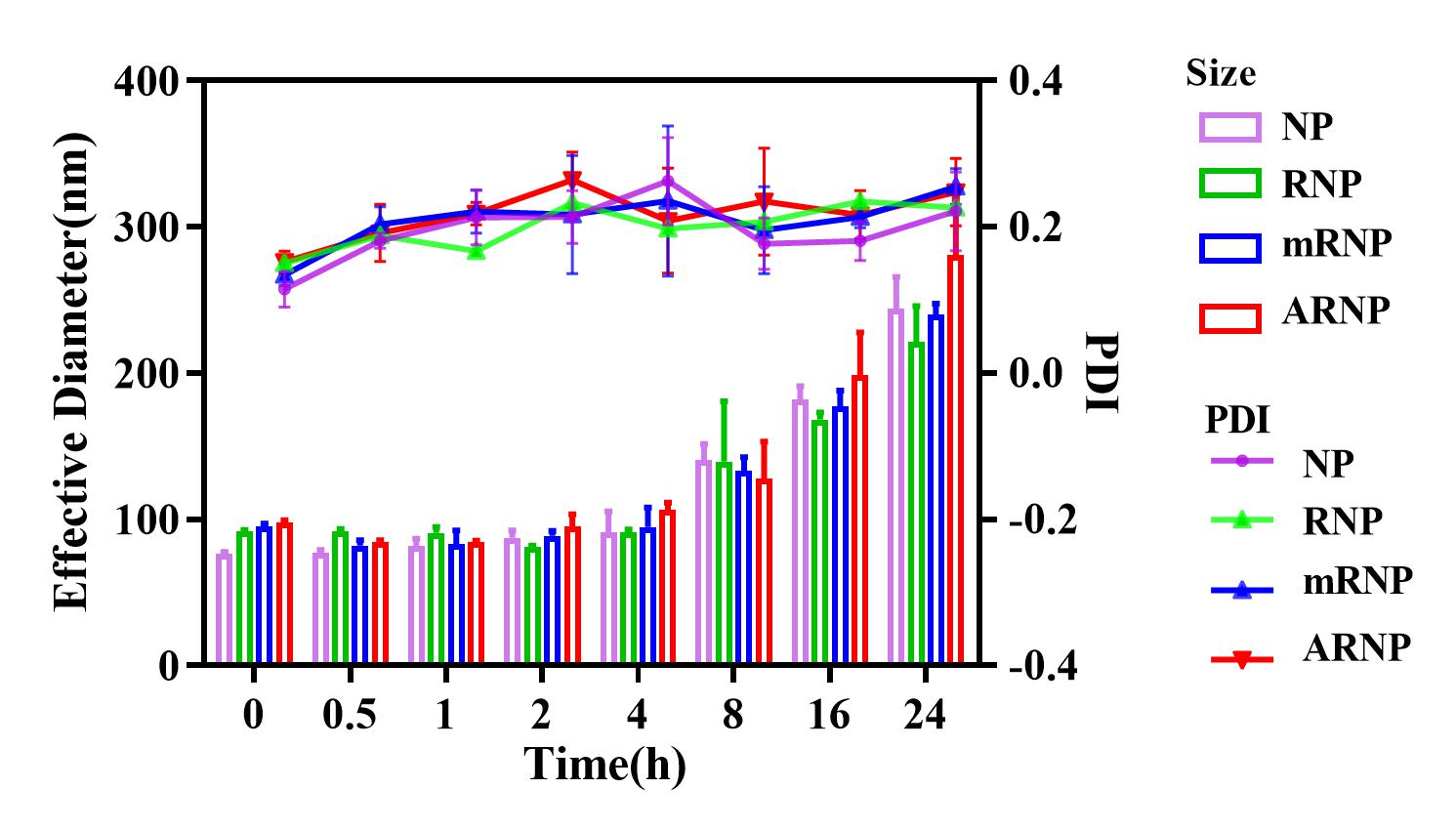
**

**Supplementary Figure 8.** The stability of nanoparticles in 10% FBS was measured by dynamic light scattering. All data are presented as the mean ± SD (n=3).

**
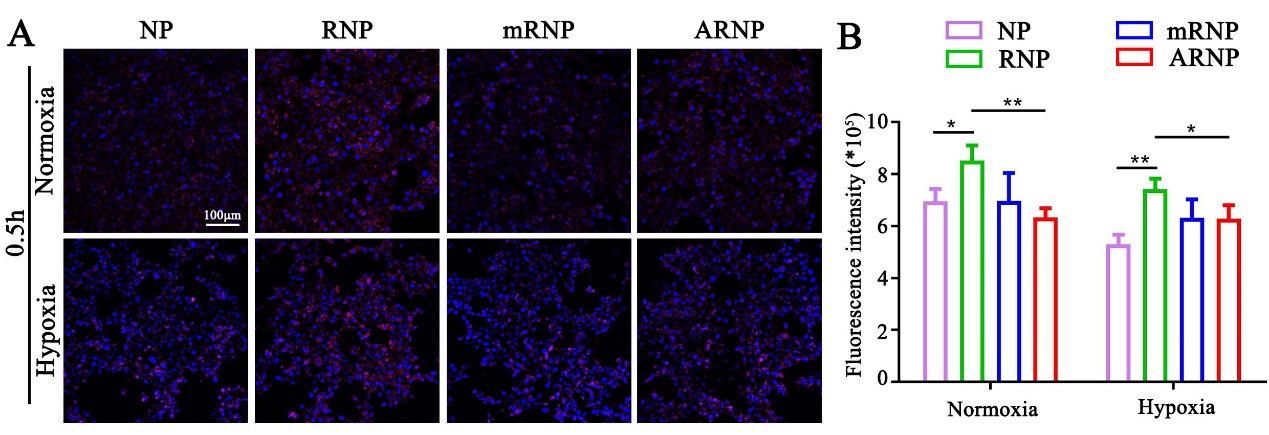
**

**Supplementary Figure 9.** Confocal imaging of cellular uptake under normoxic and hypoxic conditions at 0.5 h. All data are presented as the mean ± SD (n=3).


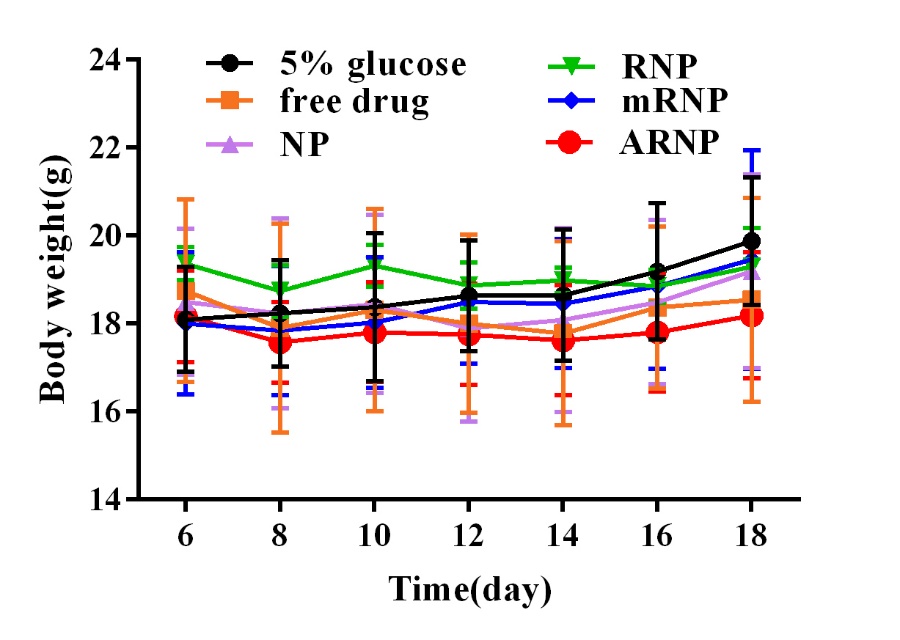


**Supplementary Figure 10.** The body weights of different treatment groups. All data are presented as the mean ± SD (n=6).


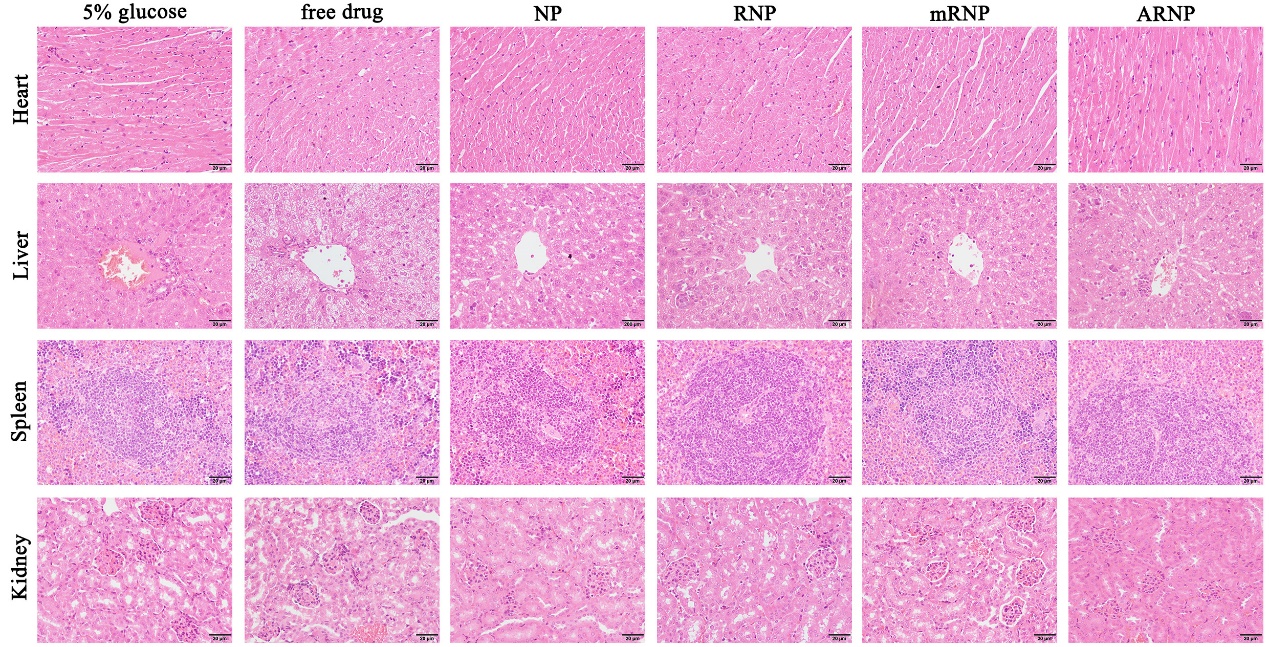


**Supplementary Figure 11.** Hematoxylin and eosin staining of the major organs (heart, liver, spleen and kidney) (scale bar = 20 μm).


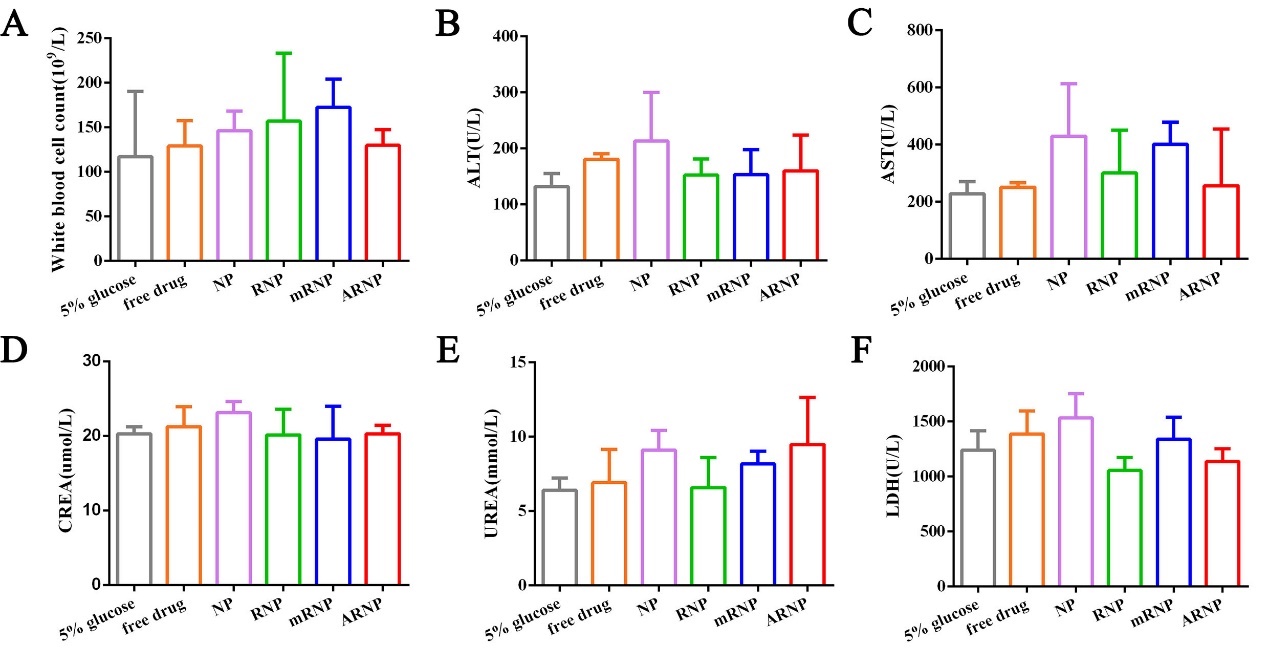


**Supplementary Figure 12.** **Detection of mouse hematological indicators.** A) While blood cell count from different treatment groups. The serum levels of ALT (B), AST (C), CREA (D), UREA (E) and LDH (F). All data are presented as the mean ± SD (n=3).

**Supplementary Table 1.** The encapsulation efficiency (EE%) and drug loading efficiency (DL%) of the prepared nanoparticles. All data are presented as the mean ± SD (n=3).

|  | **JQ1** | | **Icaritin** | |
| --- | --- | --- | --- | --- |
|  | **DL (%)** | **EE (%)** | **DL (%)** | **EE (%)** |
| NP | 14.1 ± 3.5 | 85.3 ± 6.3 | 5.8 ± 3.3 | 84.8 ± 5.7 |
| RNP | 14.7 ± 3.2 | 90.4 ± 5.8 | 7.6 ± 2.9 | 85.9 ± 4.8 |
| mRNP | 15.8 ± 4.1 | 88.7 ± 4.5 | 7.9 ± 3.4 | 83.9 ± 5.1 |
| ARNP | 15.5 ± 3.9 | 87.5 ± 4.8 | 7.5 ± 3.1 | 86.4 ± 4.6 |
